# Class I HDAC inhibitor mocetinostat induces apoptosis by activation of miR-31 expression and suppression of E2F6

**DOI:** 10.1038/cddiscovery.2016.36

**Published:** 2016-06-06

**Authors:** Q Zhang, M Sun, S Zhou, B Guo

**Affiliations:** 1 Department of Pharmaceutical Sciences, School of Pharmacy, North Dakota State University, Fargo, ND 58108, USA

## Abstract

The class I selective inhibitor of the histone deacetylases, mocetinostat, has promising antitumor activities in both preclinical studies and the clinical trials. To understand how mocetinostat induces apoptosis, we examined the effects of mocetinostat on miR-31, a proapoptotic microRNA that was previously found to be epigenetically silenced in prostate cancer. We found that miR-31 was significantly upregulated by mocetinostat in prostate cancer cells. Antiapoptotic protein E2F6, the target of miR-31, was decreased by mocetinostat treatment. When miR-31 was blocked with an inhibitor, the ability of mocetinostat to induce apoptosis was reduced. We further demonstrated that mocetinostat enhanced the activity of docetaxel in apoptosis induction. While siRNA knockdown of E2F6 sensitized cancer cells to mocetinostat-induced apoptosis, overexpression of E2F6 blocked mocetinostat-induced apoptosis. In an orthotopic xenograft model, we demonstrated that mocetinostat activated miR-31, decreased E2F6, induced apoptosis, and significantly reduced prostate cancer growth. Importantly, we found that mocetinostat also increased miR-31 expression, decreased E2F6, and induced apoptosis in the primary prostate cancer stem cells. Thus, activation of miR-31 and downregulation of E2F6 constitute an important mechanism in mocetinostat-induced apoptosis in prostate cancer.

Histone deacetylases (HDACs) are important epigenetic regulators of gene expression.^1^ These enzymes deacetylate lysines of the core histone tail and result in transcription repression.^2^ HDACs also deacetylate lysines of other proteins and regulate cellular functions ranging from proliferation and apoptosis to metastasis and angiogenesis.^3,4^ There are 18 mammalian HDACs and they are classified into four groups:^5^ class I HDACs (HDAC1, -2, -3, and -8); class II HDACs (HDAC4, -5, -6, -7, -9, and -10); class III HDACs (sirt1–7); and class IV HDAC (HDAC11). The class I HDACs are often overexpressed in various types of cancers comparing with the corresponding normal tissues and their overexpression is correlated with a poor prognosis.^6–8^ HDAC inhibitors have been developed and extensively tested in phase I–III clinical trials. However, these new agents showed minimal clinical activity in patients with solid tumors while undesired side effects are also a problem.^9^ One of the reasons for the poor performance of the early generation of the HDAC inhibitors may be because they inhibit multiple classes of HDACs, which results in toxicity and limits the achievable therapeutic doses. Mocetinostat, also known as MGCD0103, is one of the new class I selective HDAC inhibitors.^10^ Preclinical studies have demonstrated broad spectrum antitumor activities of mocetinostat against various types of cultured cell lines and tumor xenografts in nude mice.^10,11^ It has been shown that mocetinostat inhibits colon cancer cell growth by upregulating WNT ligand DKK-1 expression.^12^ In B-cell chronic lymphocytic leukemia cells, mocetinostat has been shown to induce apoptosis through decreasing antiapoptotic Mcl-1 protein and inducing Bax translocation to the mitochondria.^13^ While mocetinostat has been shown to effectively kill prostate cancer cells,^10^ the mechanisms of apoptosis induction remain poorly understood.

microRNAs (miRNAs) are small single-stranded non-coding RNAs that suppress gene expression by cleaving the target mRNAs or by inhibiting their translation.^14^ The miRNAs are initially transcribed from their encoding genes as long pri-miRNAs. The pri-miRNAs are further processed into ~60–70 nucleotide-long pre-miRNAs by Drosha RNase III endonuclease.^15^ The RNase III endonuclease Dicer then processes the pre-miRNAs to form the mature single-strand miRNAs, which are incorporated into the RNA-induced silencing complex to bind to and silence the target mRNAs. Recent studies have demonstrated important roles of miRNAs in cancer, regulating cell cycle,^16^ differentiation,^17,18^ metabolism,^19^ invasion and metastasis,^20^ as well as apoptosis.^21^ Recently, we have reported that miR-31 was significantly downregulated in prostate cancer cells and its downregulation resulted in overexpression of antiapoptotic protein E2F6 and resistance to apoptosis.^22^ Studies by Lin *et al.*^23^ have shown that miR-31 is epigenetically silenced in prostate cancer by promoter hypermethylation and histone H3K27 trimethylation. Since the HDACs are often involved in DNA methylation-mediated gene silencing,^24^ we tested whether mocetinostat can activate miR-31 expression. We found that mocetinostat activated miR-31 expression and downregulated its target, the antiapoptotic protein E2F6 both *in vitro* and *in vivo* in an orthotopic xenograft model. These findings identified a novel mechanism that contributes to mocetinostat-induced apoptosis in prostate cancer.

## Results

### Mocetinostat induces apoptosis in prostate cancer cells

**Figure 1:**
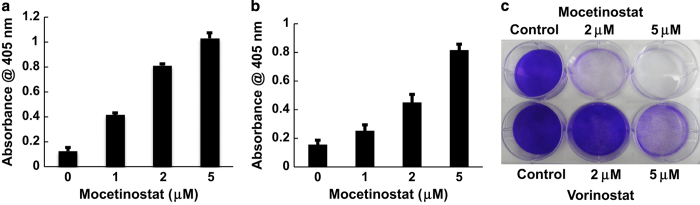
Mocetinostat induces apoptosis in prostate cancer cells. DU-145 cells (**a**) and PC-3 cells (**b**) were treated with various doses of mocetinostat for 24 h and apoptosis was analyzed using the Cell Death Detection Elisa^PLUS^ kit as described in Materials and Methods. (**c**) DU-145 cells were treated with various doses of mocetinostat or vorinostat for 72 h. The colonies were fixed with paraformaldehyde and stained with 0.05% crystal violet. All experiments have been repeated three times; data shown are mean values +S.D.

We determined whether mocetinostat can induce apoptosis in prostate cancer cells. As shown in Figure 1a, mocetinostat induced significant levels of apoptosis in DU-145 cells in a dose-dependent manner. Induction of apoptosis was determined by the cell death ELISA assay measuring mono- and oligonucleosomes in the lysates of apoptotic cells. Similarly, mocetinostat also induced significant levels of apoptosis in PC-3 cells (Figure 1b). We compared the antitumor activities of mocetinostat and vorinostat (suberanilohydroxamic acid),^25^ an FDA-approved non-selective HDAC inhibitor. Mocetinostat was significantly more potent than vorinostat in prostate cancer suppression (Figure 1c).

### Mocetinostat induces miR-31 expression and downregulates E2F6

**Figure 2:**
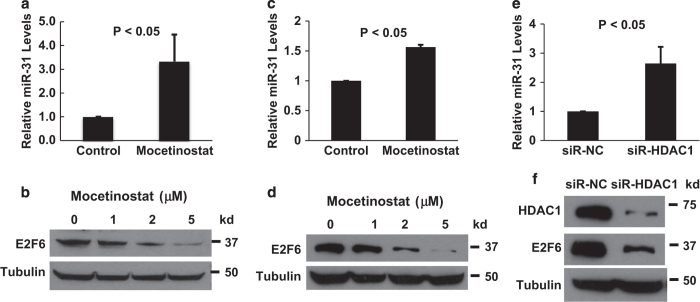
Mocetinostat activates miR-31 expression in prostate cancer cells. (**a**) DU-145 cells and (**c**) PC-3 cells were treated with 5 μM mocetinostat for 24 h. Total RNA was isolated from untreated (control) and mocetinostat-treated cells and real-time PCR analysis was performed as described in Materials and Methods. (**b**) DU-145 and (**d**) PC-3 cells were treated with various doses of mocetinostat for 24 h. Western blotting was performed with anti-E2F6 and antitubulin antibodies. (**e** and **f**) DU-145 cells were transfected with negative control or HDAC1 siRNAs. Real-time PCR and western blotting were performed as described in (**a**–**d**). The experiments have been repeated three times; data shown are mean values +S.D.

**Figure 3:**
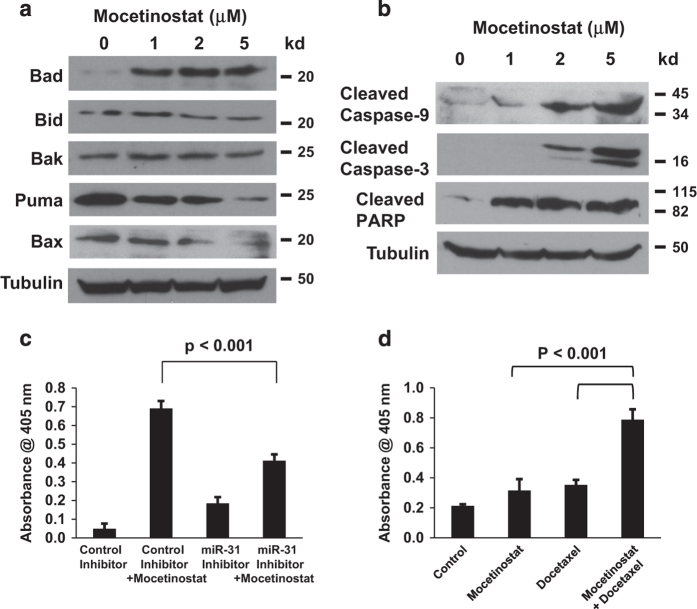
Mocetinostat increases Bad expression and enhances the activity of docetaxel. (**a** and **b**) DU-145 cells were treated with various doses of mocetinostat for 24 h. Western blotting was performed using the indicated antibodies. (**c**) DU-145 cells were transfected with negative control or anti-miR-31 inhibitor, followed by treatment with 1 μM mocetinostat for 24 h. Apoptosis was analyzed using the Cell Death Detection Elisa^PLUS^ kit. (**d**) DU-145 cells were treated with 1 μM mocetinostat or 10 nM docetaxel as a single agent or in combination for 24 h. Apoptosis was analyzed using the Cell Death Detection Elisa^PLUS^ kit. The experiments have been repeated three times; data shown are mean values +S.D.

We recently demonstrated that the downregulation of miR-31 contributes to apoptosis resistance in prostate cancer cells.^22^ Since miR-31 was shown to be repressed by epigenetic mechanisms in prostate cancer,^23^ we hypothesized that mocetinostat may activate miR-31 expression. The effects of mocetinostat on miR-31 expression were determined by real-time PCR. As shown in Figures 2a and c, mocetinostat significantly induced miR-31 expression in both DU-145 and PC-3 cells. We have previously shown that miR-31 targets E2F6,^22^ which is a potent antiapoptotic protein that can inhibit UV- and hypoxia-induced apoptosis.^26,27^ As a result of miR-31 induction, mocetinostat significantly decreased E2F6 protein in DU-145 and PC-3 cells (Figures 2b and d). To determine whether HDAC inhibition by mocetinostat is responsible for the activation of miR-31, we used siRNA to specifically knock down HDAC1. As shown in Figures 2e and f, siRNA knockdown of HDAC1 activated miR-31 expression and decreased E2F6 protein. To further understand the mechanism of mocetinostat-induced apoptosis, we determined the effects of mocetinostat on the expression of the proapoptotic members of the Bcl-2 family proteins.^28^ Interestingly, Bad was significantly increased by mocetinostat treatment while the expression levels of the other proteins were either reduced (Puma, Bid, and Bax) or unchanged (Bak) (Figure 3a). Since Bad is a key proapoptotic protein that triggers the intrinsic pathway of apoptosis,^29,30^ we examined the effects of mocetinostat on the caspases. As shown in Figure 3b, mocetinostat treatment significantly increased the levels of activated (cleaved) caspase-9 and caspase-3. The cleaved products of PARP, substrates of the caspases, were also increased by mocetinostat.

### miR-31 plays a critical role in mocetinostat-induced apoptosis and mocetinostat enhances the activity of docetaxel

To determine the role of miR-31 in mocetinostat-induced apoptosis, we used an miR-31 inhibitor to block the activity of miR-31 in DU-145 cells. As shown in Figure 3c, the ability of mocetinostat to induce apoptosis was reduced when miR-31 was inhibited. In metastatic castration-resistant prostate cancer, docetaxel is the only approved treatment and it only has limited efficacy. To determine whether mocetinostat can enhance the antitumor activity of docetaxel, we treated DU-145 cells with a single agent of mocetinostat and docetaxel, or the combination of the two agents. As shown in Figure 3d, mocetinostat significantly enhanced apoptosis induction by docetaxel.

### E2F6 regulates mocetinostat-induced apoptosis in prostate cancer cells

**Figure 4:**
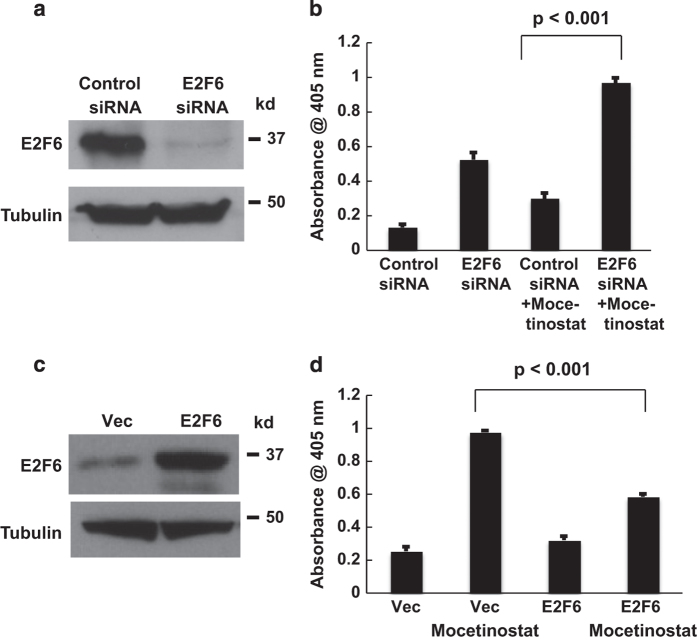
E2F6 regulates mocetinostat-induced apoptosis. (**a**) DU-145 cells were transfected with the negative control or E2F6-targeting siRNAs. At 48 h after siRNA transfection, cell lysates were analyzed by western blotting with the indicated antibodies. (**b**) DU-145 cells were transfected with the negative control or E2F6 siRNAs. At 24 h after siRNA transfection, cells were treated with 1 μM mocetinostat for additional 24 h. Apoptosis was measured by Cell Death Detection Elisa^PLUS^ analysis as described in Materials and Methods. The experiments have been repeated three times; data shown are mean values+S.D. (**c**) DU-145 cells were transfected with the empty expression vector or pcDNA3-HA-E2F6. At 24 h after transfection, E2F6 overexpression was confirmed by western blot. (**d**) DU-145 cells were transfected with the empty expression vector or pcDNA3-HA-E2F6. At 24 h after transfection, cells were treated with 5 μM of mocetinostat for additional 24 h and apoptosis was measured by Cell Death Detection Elisa^PLUS^ analysis as described in Materials and Methods. The experiments have been repeated three times; data shown are mean values +S.D.

To determine the role of E2F6 in mocetinostat-induced apoptosis, we used siRNA to knock down E2F6 in DU-145 cells. As shown in Figure 4a, E2F6 protein expression was significantly reduced by siRNA treatment. Knockdown of E2F6 sensitized DU-145 cells to apoptosis induced by mocetinostat (Figure 4b). We expressed E2F6 exogenously to determine if E2F6 can contribute to apoptosis resistance in prostate cancer cells. Overexpression of E2F6 was confirmed by western blotting (Figure 4c). When treated with mocetinostat, DU-145 cells overexpressing E2F6 were significantly more resistant to drug-induced apoptosis, comparing with empty vector-transfected cells (Figure 4d).

### Mocetinostat induces miR-31 expression and activates apoptosis *in vivo* in an orthotopic tumor xenograft model

**Figure 5:**
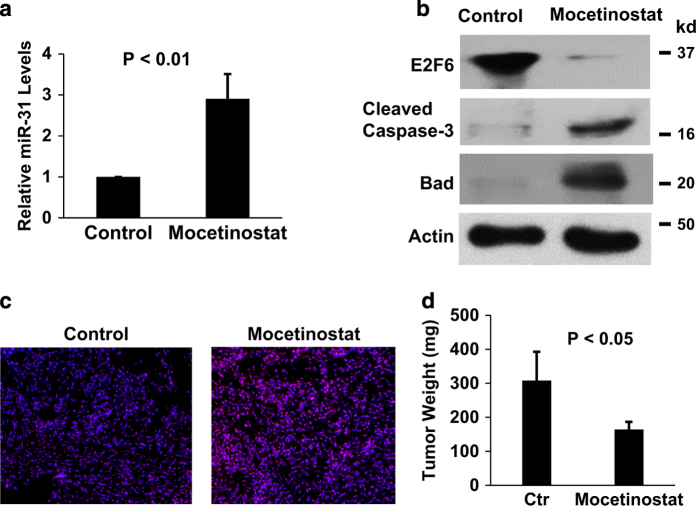
Mocetinostat activates miR-31 and induces apoptosis in an orthotopic xenograft model. (**a**) Orthotopic tumor xenografts were established in nude mice using DU-145 cells as described in Materials and Methods. In the control group (5 mice), mice were given PBS. In the treatment group (5 mice), mice were given 100 mg/kg mocetinostat in drinking water daily.^10^ Total RNA was isolated from untreated (control) and mocetinostat-treated tumors and real-time PCR analysis was performed as described in Materials and Methods. (**b**) Total protein was isolated from the tumors; western blotting was performed with the indicated antibodies. (**c**) *In vivo* apoptosis induction was detected by TUNEL assay as described in Materials and Methods. Red: BrdU; blue: DAPI. (**d**) At the end of mocetinostat treatment, mice were killed and tumors were removed and weighted.

To determine whether miR-31 activation plays an important role in mocetinostat-induced apoptosis *in vivo*, we established an orthotopic xenograft model in which the prostate cancer was implanted into the prostate of nude mice. After treatment with mocetinostat, we observed *in vivo* activation of miR-31, downregulation of E2F6, increased expression of Bad, and activation of caspase-3 (Figures 5a and b). As a result, significant level of apoptosis was induced in the tumors and orthotopic tumor growth was suppressed by nearly 50% (Figures 5c and d). We did not observe any toxicity in the mice (such as loss of body weight, etc).

### Mocetinostat induces miR-31 expression and activates apoptosis in primary prostate cancer stem cells

**Figure 6:**
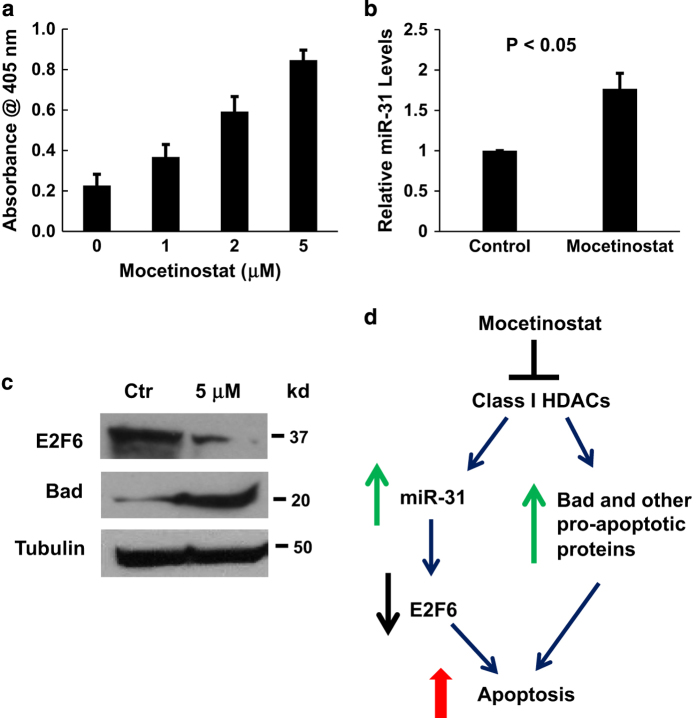
Mocetinostat induces apoptosis in the primary prostate cancer stem cells. (**a**) Prostate cancer stem cells were treated with various doses of mocetinostat for 24 h and apoptosis was analyzed using the Cell Death Detection Elisa^PLUS^ kit. (**b**) Prostate cancer stem cells were treated with 5 μM mocetinostat for 24 h. Total RNA was isolated and real-time PCR analysis was performed as described in Materials and Methods. (**c**) Prostate cancer stem cells were treated with 5 μM of mocetinostat for 24 h. Western blotting was performed with the indicated antibodies. The experiments have been repeated three times; data shown are mean values+S.D. (**d**) Schematic illustration of the role of miR-31 in mocetinostat-induced apoptosis. By inhibiting the class I HDACs, mocetinostat activates miR-31 expression and subsequently decreases E2F6. In the meantime, mocetinostat also increases the proapoptotic protein Bad. As a result, apoptosis is triggered in the prostate cancer cells.

It is postulated that cancer stem cells mediate tumor formation, metastasis, and resistance to chemotherapy.^31^ Thus, it is critical to identify drugs that can eliminate cancer stem cells.^32^ We tested if mocetinostat is effective against prostate cancer stem cells. As shown in Figure 6a, mocetinostat induced significant levels of apoptosis in patient-derived primary prostate cancer stem cells. Furthermore, mocetinostat increased miR-31 expression (Figure 6b), and decreased E2F6 protein (Figure 6c) in the prostate cancer stem cells. The level of proapoptotic protein Bad was increased by mocetinostat (Figure 6c).

## Discussion

Mocetinostat is a class I selective HDAC inhibitor and has promising antitumor activities in preclinical studies.^10,11^ More importantly, recent clinical trials have demonstrated excellent response of mocetinostat against myelodysplastic syndrome and relapsed Hodgkin's lymphoma, with acceptable safety profiles.^33^^,^^34^ However, a phase II study also found that mocetinostat has limited efficacy as a single agent in relapsed and refractory chronic lymphocytic leukemia.^35^ Thus, it is important to understand the mechanism of mocetinostat-induced apoptosis in order to improve its efficacy in cancer therapy and overcome drug resistance. Our findings in this report serve for this purpose by helping to understand the molecular basis of mocetinostat-induced apoptosis. We have previously shown that in prostate cancer cell lines derived from advanced metastatic cancers, miR-31 is downregulated to contribute to the resistance to chemotherapy-induced apoptosis.^22^ Here, we demonstrated that mocetinostat activates the expression of miR-31, which in turn decreases the antiapoptotic protein E2F6. We also found that mocetinostat increases the expression of proapoptotic protein Bad. Our data have established a mechanistic model to explain how mocetinostat induces apoptosis in prostate cancer (Figure 6d). By inducing miR-31 to decrease E2F6 while in the meantime increasing Bad, mocetinostat tipped the balance between the antiapoptotic and proapoptotic proteins,^36^ resulting in increased apoptosis.

Evidences are emerging that the prostate cancer stem cells may play important roles in resistance to castration^37^ and chemotherapy.^31^ Recently, it has been shown that mocetinostat induces cell cycle arrest and apoptosis in colon-cancer-initiating cells.^12^ Our data suggest that mocetinostat can efficiently induce apoptosis in the primary prostate cancer stem cells through a mechanism involving miR-31 and E2F6 (Figure 6). Thus, mocetinostat is an effective drug for eliminating the prostate cancer stem cells.

Azacitidine can activate the expression of epigenetically silenced genes by inhibiting DNA promoter methylation.^38^ Moreover, recent clinical trials have demonstrated excellent results when azacitidine and mocetinostat are used together in cancer therapy.^33^ It will be interesting to test if azacitidine can act with mocetinostat synergistically to activate miR-31 and induce apoptosis in prostate cancer cells, since the two drugs target different mechanisms of miR-31 silencing.

In current clinical trials of mocetinostat and other HDAC inhibitors, HDAC activity inhibition and histone acetylation are used as biomarkers to measure pharmacodynamics responses. Our findings in this study have identified new parameters (miR-31, E2F6, Bad) that may be used to monitor tumor response to mocetinostat. These apoptosis regulators may be useful to predict how efficiently mocetinostat can kill the prostate cancer cells.

## Materials and Methods

### Cells and transfection

The cell lines PC-3 and DU-145 were purchased from American Type Culture Collection (Manassas, VA, USA). Cells were cultured in RPMI1640 media containing 10% FBS. Patient-derived human prostate cancer stem cells were purchased from CELPROGEN (San Pedro, CA, USA) and cultured following the manufacturer’s protocol. For transient transfection, plasmids were transfected into cells using Lipofectamine Plus Reagent (Invitrogen, Grand Island, NY, USA) following the manufacturer’s protocol. E2F6 siRNA (Ambion, Grand Island, NY, USA; Cat # 4185), HDAC1 siRNA (Ambion; Cat # s73) and anti-miR-31 inhibitor (Ambion, Cat # AM11465) were transfected into cells using X-treme GENE siRNA transfection reagent (Roche, Indianapolis, IN, USA) following the manufacturer’s protocol.

### Drugs and chemicals

Mocetinostat was purchased from Selleckchem (Houston, TX, USA). Vorinostat was purchased from Biovision (Mountain View, CA, USA).

### Plasmid construction

The full-length *E2F6* cDNA was obtained by PCR using an EST clone as a template and constructed into a pcDNA3-HA vector.

### Western blot analysis

Cells were lysed in RIPA buffer (1% NP-40, 0.5% sodium deoxycholate, 0.1% SDS in PBS). Complete protease inhibitor cocktail (Roche) was added to lysis buffer before use. Protein concentration was determined by Bio-Rad DC protein assay (Bio-Rad). Protein samples were subjected to SDS-PAGE and transferred onto nitrocellulose membrane. The membrane was blocked in 5% non-fat milk in PBS overnight and incubated with primary antibody and subsequently with appropriate horse radish peroxidase-conjugated secondary antibody. Signals were developed with ECL reagents (Pierce, Rockford, IL, USA) and exposure to X-ray films. Anti-β-tubulin and anti-E2F6 antibodies were purchased from Santa Cruz Biotechnology (Dallas, TX, USA). Cleaved caspase-9, cleaved caspase-3, cleaved PARP, HDAC1, Bad, Bid, Bak, Puma, and Bax antibodies were purchased from Cell Signaling (Danvers, MA, USA).

### Real-time PCR

The miRNA expression was measured by real-time PCR using TaqMan MicroRNA assays (Cat # TM1100 for miR-31) from Applied Biosystems (Foster city, CA, USA). Total RNA was isolated from cells using *mir*Vana miRNA Isolation Kit (Ambion). Five micrograms of total RNA was used in reverse transcription reaction. The cDNAs were used as templates to perform PCR on an Applied Biosystems 7500 Real-time PCR System following the manufacturer’s protocol. Relative miRNA expression levels were calculated using 18S RNA as reference.

### Detection of apoptosis

For *in vitro* apoptosis detection, the Cell Death Detection Elisa^PLUS^ kit (Roche) was used to detect apoptosis following the manufacturer’s protocol. This assay determines apoptosis by measuring mono- and oligonucleosomes in the lysates of apoptotic cells. The cell lysates were placed into a streptavidin-coated microplate and incubated with a mixture of anti-histone-biotin and anti-DNA-peroxidase. The amount of peroxidase retained in the immunocomplex was photometrically determined with ABTS as the substrate. Absorbance was measured at 405 nm. For *in vivo* apoptosis detection, the *in situ* BrdU-Red DNA Fragmentation (TUNEL) Assay Kit (Abcam, Cambridge, MA, USA) was used following the manufacturer’s protocol. This assay utilizes Br-dUTP (bromolated deoxyuridine triphosphate nucleotides), which is readily incorporated into DNA strand breaks. The Br-dUTP sites are identified by a red fluorescence labeled anti-BrdU monoclonal antibody.

### Crystal violet staining

The cells were treated with various doses of mocetinostat or the same doses of vorinostat for 72 h. The colonies were fixed with paraformaldehyde and stained with 0.05% crystal violet.

### Orthotopic tumor xenograft in nude mice

Six- to eight-week-old male nude mice (Nu/Nu) were purchased from Charles River (Wilmington, MA, USA). The mice were maintained in sterile conditions using the Innovive IVC System from Innovive (San Diego, CA, USA), following the protocol approved by the Institutional Animal Care and Use Committee of North Dakota State University. Orthotopic tumor xenografts were established by injection of 3×10^5^ cancer cells in 50 μl serum-free media (containing 50% Matrigel) into the prostate of the mice, after an incision was made 3 mm above the pubic symphysis and the bladder and seminal vesicles were carefully lifted to expose the dorsal prostate. Mocetinostat was dissolved in PBS acidified with 0.1 N HCl and dosed p.o. as solutions daily. PBS was given p.o. to the control group.

### Statistical analysis

Differences between the mean values were analyzed for significance using the unpaired two-tailed Student’s test for independent samples; *P*⩽0.05 was considered to be statistically significant.
